# Current state of preoperative embolization for spinal metastasis – A survey by the EANS spine section

**DOI:** 10.1016/j.bas.2023.102712

**Published:** 2023-11-10

**Authors:** Stefan Motov, Felix Stengel, Florian Ringel, Oliver Bozinov, Martin N. Stienen

**Affiliations:** a Department of Neurosurgery, Kantonsspital St. Gallen & Medical School of St. Gallen, St.Gallen, Switzerland; b Spine Center of Eastern Switzerland, Kantonsspital St. Gallen & Medical School of St. Gallen, St.Gallen, Switzerland; c University Hospital Mainz & Johannes Gutenberg-University Mainz, Mainz, Germany

**Keywords:** Spine tumor, Metastasis, Preoperative embolization, Angiography, Blood loss, Complications

## Abstract

**Introduction:**

Preoperative embolization (PE) for spinal metastasis can be used to reduce tumor blood supply in selected patients. The decision whether and when to perform PE varies largely among spine surgeons and centers.

**Research question:**

The aim was to understand the current decision-making process in European spine centers.

**Material and methods:**

The European Association of Neurosurgical Societies (EANS) spine section designed a 13-item online survey. It was distributed to neurosurgical residents and board-certified neurosurgeons between 7th of February and May 5, 2023.

**Results:**

We analyzed 120 survey responses. Most participants were board-certified neurosurgeons (71%) or residents (26%) in university hospitals (76%). Routinely performed PE was stated not a common practice in 62%. Of those using PE, 25% indicated to perform it in selected cases requiring vertebral body replacement. Reasons for not performing PE included lack of time (44%), unclear benefits (25%), no significant bleeding without PE (19%), and significant bleeding despite PE (8%). Most participants opted for PE < 24h before surgery, but in a separate anesthesia (54%). More experienced participants were more likely to observe reduced blood loss (BL) after PE (p = 0.014). The most common reported complications were neurological deterioration due to spinal cord infarction (n = 15) and swelling due to tumor necrosis (n = 13).

**Discussion and conclusions:**

PE is still not a routine among European spine surgeons and is considered mostly for elective cases with hypervascularized tumors scheduled in a separate anesthesia <24h before tumor resection. Most participants noted reduced intraoperative BL, but also a risk of procedure-related complications.

## Highlights

• Survey on current state of preoperative embolization for spinal metastasis.
• Preoperative embolization appears to be a non-routine procedure for mainly hypervascularized spinal metastasis.
• Optimal time frame for PE < 24 h before surgery in a separate anesthesia.
• PE is associated with a reduction of EBL but also with a non-negligible rate of observed procedure-related complications.

## 1 Introduction

Preoperative embolization (PE) for spinal metastasis is a procedure aiming to reduce the blood supply in tumor masses and alleviate pain in a selected group of patients. In the context of tumor resection prior studies demonstrated significant reduction of perioperative bleeding and postoperative transfusions, especially in hypervascularized lesions with improved outcome and safety of the subsequent surgical procedure (Gao et al., 2021; Kato et al., 2012; Wahood et al., 2021). Approximately 60% of spinal metastases are hypervascularized (Truumees et al., 2010) (e.g. renal cell carcinoma, thyroid carcinoma etc.) and although embolization is not effective in controlling epidural venous plexus bleeding, it enables a better visualization and decreases estimated intraoperative blood loss (EBL) and surgical times through a reduction in patent arterial feeders. In cases with marginal resection, PE may even be beneficial to reduce tumor recurrence (Truumees et al., 2010).

In general, PE have been described as safe procedures with low complications rate in the current literature (Houten et al., 2020). However, the decision whether and when to perform PE varies largely among spine surgeons and centers (Wahood et al., 2021; Kato et al., 2013; Yuh, 2023).

We hence designed a survey to explore the current state of PE for spinal metastasis across spine centers in the European Association of Neurosurgical Societies (EANS) member states. We aimed to explore the decision-making process, the framework, frequency of interventions and resulting complications among spine specialists and units.

## 2 Methods

### 2.1 Web-based survey and distribution

Board-certified specialists and residents affiliated with the EANS were invited to fill out a 13-question online questionnaire between 7th of February and May 5, 2023. The survey was designed using the SurveyMonkey platform (https://www.surveymonkey.com) and distributed by the EANS spine section email platform. We used our personal networks to colleagues, friends, and national societies (e.g., Swiss Young Neurosurgeons, German Society of Neurosurgery) as well as social media to additionally promote the survey among the neurosurgical community.

### 2.2 Survey design

The first questions contained demographic information of the participants and general characteristics, while the remaining explored the frequency of embolization procedures for spinal metastases, the decision-making process behind the indication for PE, as well as the time interval between embolization and subsequent surgical treatment. Participants stated how often they experienced complications with PE and if they believed or have evaluated that PE reduced the EBL. The complete survey can be found as Supplemental Nr. 1. Survey results were checked for duplicates and missing data.

### 2.3 Statistical analysis

All statistical analyses and generation of all graphs were performed using StataSE 15 (StataCorp. 2017. Stata Statistical Software: Release 15. College Station, TX: StataCorp LLC). Descriptive statistics were employed, describing the responses as count (percent) and mean [standard deviation (SD)]. Graphical illustrations of results were used to explore relationships. We used logistic regression to analyze influence of co-variables on dichotomous questions. Paired-sample t-tests, multinominal, and ordinal logistic regression analyses were applied to explore the subjective value of different workplaces and the surgical experience based on number of annual surgeries on how routinely PEs have been performed and if complications occurred. Surgical experience was defined depending on the number of spinal stabilization procedures for vertebral metastases per year, using 10 procedures as cut-off. Results were considered significant at p-values <0.05.

## 3 Results

### 3.1 Survey responses

The estimated number of specialists who were invited to respond to the survey were 3000. No reminder Emails were sent out to respect the EANS members’ decision of non-participation. We received 121 responses (response rate = 4 %), of which one was excluded for missing relevant data. None of the datasets were removed as duplicate. Finally, a total of 120 survey responses were considered for analysis.

### 3.2 Demographics and characteristics of participants

All demographic data are summarized in Table 1. The mean age of participants was 39 (SD ± 9.9) years. The female (n = 24, 20%) to male (n = 96, 80%) ratio was 1:4. Most respondents were board-certified neurosurgeons (n = 85; 70.8%) or neurosurgical residents in advanced training (n = 31, 25.8%). Three board-certified orthopedic surgeons (2.5%) and one medical student (0.8 %) also participated. Three out of four participants were employed at university hospitals (n = 91, 75.8 %), followed by specialists at non-university or public hospitals (n = 22, 18.3 %) and private hospitals (n = 7, 5.8%). The annual number of spinal stabilization procedures for vertebral metastases was low (max. 5 surgeries per year - n = 39, 32.5%) to moderate (5–30 surgeries per year - n = 71, 59.3%) among most respondents. Only ten participants (8.3%) stated a higher annual number >30 procedures per year.

**Table 1:** Demographic data and Participant characteristics.

	Number (Proportion)
**Gender**	
Female	24 (20.0 %)
Male	96 (80.0 %)
Missing	1 (0.8 %)
**Professional degree**	
Board-certified in neurosurgery	85 (70.8 %)
Board-certified in orthopedic surgery	3 (2.5 %)
Board-certified in trauma surgery/traumatology	0 (0 %)
Resident in advanced training	31 (25.8 %)
Other	1 (0.8 %) – Medical student
Missing	1 (0.8 %)
**Type of hospital**	
University hospital	91 (75.8 %)
Public, non-university hospital	22 (18.3 %)
Private hospital	7 (5.8 %)
Medical practice	0 (0 %)
Other (please specify)	0 (0 %)
Missing	1 (0.8 %)
**Average number of spinal stabilization procedures personally performed for vertebral metastases per year (Experience level)**	
<5 surgeries	39 (32.5 %)
5–10 surgeries	32 (26.7 %)
10-20 surgeries	26 (21.7 %)
20-30 surgeries	13 (10.8 %)
>30 surgeries	10 (8.3 %)

### 3.3 Frequency and rationale for PE in patients with spinal metastasis

**Fig. 1:**
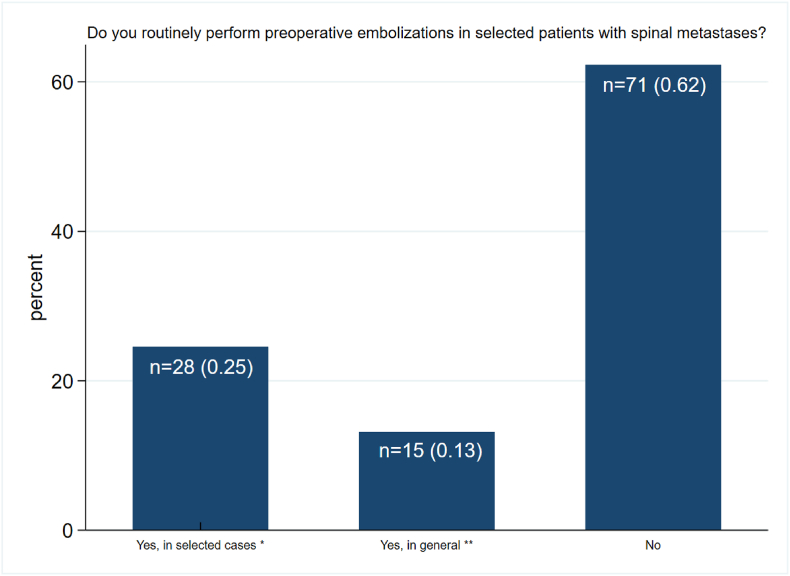
Routine use of preoperative embolization (PE) in patients with spinal metastasis: The bar chart shows the answers to question “Do you routinely perform preoperative embolizations in selected patients with spinal metastases?”. The absolute numbers (n) and percentages are shown in the bar. Of the respondents, 28 (0.25) indicated, to perform PE, but only when vertebral body resection/replacement is necessary (*). Routine PE in general before any elective surgical treatment (including tumor decompression alone, "separation surgery" without vertebral body replacement, etc.) was reported by 15 participants (0.13) (**). The majority stated that they did not perform routine pE (n = 71, 0.62). Missing: n = 6.

On the question “Do you routinely perform preoperative embolization in patients with spinal metastases?”, n = 71 patients (62.3%) stated that this is not common practice. Amongst those using PE, n = 28 (24.6%) indicated to perform embolization only in selected cases where vertebral body resection and replacement is necessary and n = 15 (13.2%) selected use of PE before any elective surgical treatment (including tumor decompression, "separation surgery" without vertebral body resection, etc.). Altogether, less than half of all participants (n = 43; 37.7%) indicated to perform PE on a routine basis (Fig. 1). Six participants skipped this question.

Of those who stated to perform PEs routinely, the majority (n = 39/43; 91%) specified their use for hypervascularized tumors, e.g., renal cell carcinoma or thyroid carcinoma. Four participants (9.1%) indicated using PEs in cases of tumor recurrence or pre-irradiated operative field and two participants (4.5%) in general for all dignities.

The possible influence of the work setting (p = 0.35) and the experience level (p = 0.28) on the use of routine PE was analyzed with a multinomial logistic regression analysis without revealing significant correlation.

### 3.4 Reasons for not using PE in patients with spinal metastases

**Fig. 2:**
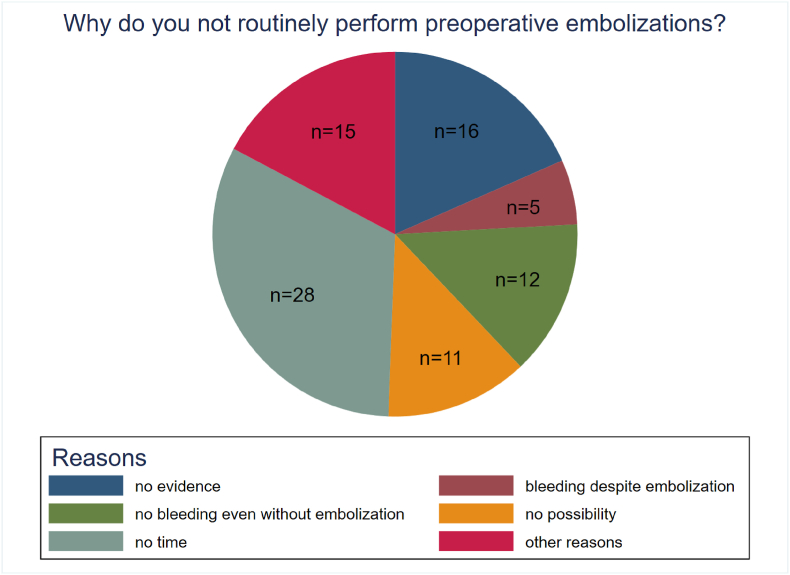
Why do you not routinely perform preoperative embolization (PE): Pie chart shows reported reasons why routine preoperative embolization is not performed. The majority (n = 28, 43.8%) answered they usually operate on emergency cases without suitable time for PE. Other participants specified that there was no clear evidence regarding the benefit (n = 16, 25%), that there was no significant bleeding without PE anyway (n = 12, 18.8 %) or that there was significant amount of bleeding despite PE (n = 5, 7.8 %). Eleven respondents (17.2%) stated there was no possibility for PE in their institutions. Fifteen respondents declared they do not routinely perform PEs for other reasons, e.g., only in selected cases (n = 8, 12.5%), reluctancy of the interventionalists (n = 4, 6.3%), non-existing emergency radiologist service (n = 2, 3.1%) or due to potential risk for neurological deterioration (1, 1.5%). 57 participants skipped the question.

We asked those who stated to not perform PEs routinely for their reasons. The majority (n = 28, 43.8%) answered they usually operate on emergency cases without suitable time for PE. Other participants specified that there was no clear evidence regarding the benefit (n = 16, 25%), that there was no significant bleeding without PE anyway (n = 12, 18.8%) or that there was significant amount of bleeding despite PE (n = 5, 7.8%). Eleven respondents (17.2%) stated there was no possibility for PE in their institutions. Fifteen respondents declared they do not routinely perform Pes for other reasons, e.g., only in selected cases (n = 8, 12.5%), reluctancy of the interventionalists (n = 4, 6.3%), non-existing emergency radiologist service (n = 2, 3.1%) or due to potential risk for neurological deterioration (1, 1.5%). 57 participants skipped the question (Fig. 2).

The availability of PE was associated with the type of work setting, revealing a higher availability in public, non-university hospitals (OR 4.8, 95% CI 1.2–18.9, p = 0.03) than in university hospitals. However, there was a low rate of responses from public (n = 22) and very low rate from private clinics (n = 7), compared with participants from university hospitals (n = 91), which may have influenced these findings.

### 3.5 Imaging diagnostics and timing of PE

Key factors influencing the decision-making process for the indication of PE included most commonly the suspected tumor histopathology (n = 49; 48%) and preoperative MRI and/or CT features (n = 37; 36.3%). Imaging appearance on preoperative MR- or CT-angiography were important criteria for 24 participants (23.5%); further 24 participants (23.5%) indicated considering diagnostic subtraction angiography (DSA). Fifteen respondents (14.6%) considered preoperative anemia or anticoagulation status as relevant criteria for requesting PE. 19 participants skipped the question.

**Fig. 3:**
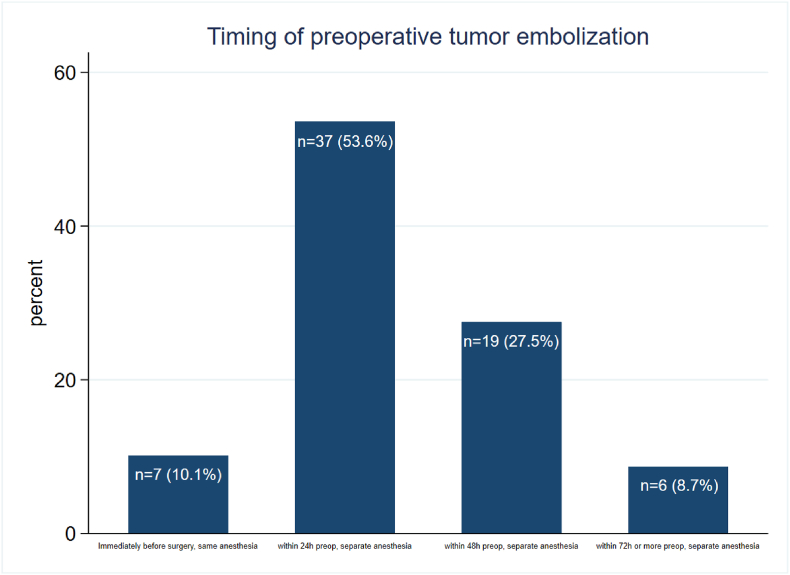
Timing of preoperative embolization (PE) with respect to the subsequent surgery: The bar graph shows that most participants performed PE 24 h preoperatively under separate anesthesia (n = 37, 0.536), followed by 19 participants (0.275) with PE 48h preoperatively. PE 72 h before surgery is reported by 6 participants (0.087). Immediate preoperative PE under anesthesia itself is reported by 7 participants (0.101). Missing: n = 19.

Regarding the timing of PE for subsequent surgery, most participants specified they prefer requesting the intervention <24 h before surgery in a separate anesthesia (n = 37, 53.6%) (Fig. 3). Seven respondents (10.1%) stated they request the embolization immediately before surgery in the same anesthesia, while the rest indicated they prefer to have it either 48 h (n = 21, 27.5 %) or 72 h (n = 6, 8.7%) before surgery in a separate anesthesia. 19 participants skipped the question.

**Fig. 4:**
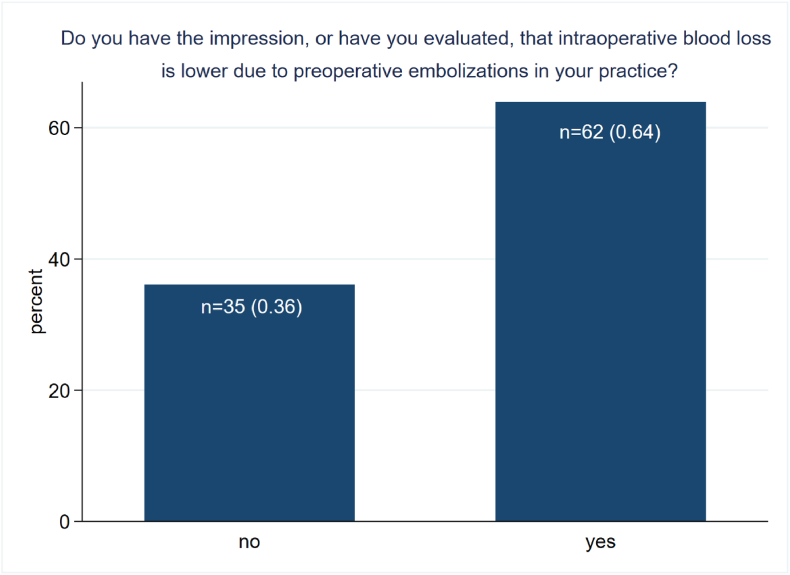
Impression/Evaluation of lower intraoperative blood loss (EBL) due to preoperative embolization (PE) in daily practice. *The bar chart shows the answers to the question: “Do you have the impression, or have you evaluated, that intraoperative blood loss is due to preoperative embolizations is lower in your practice?”. The majority of respondents (n=62, 0.64) answered this question in the affirmative. Missing: n=23.*

### 3.6 Intraoperative blood loss and complications

In the estimated blood loss (EBL) question, 62 respondents (63.9 %) had the impression, or have evaluated, that the EBL is lower after PE (Fig. 4). In contrast, 35 respondents (36.1 %) were not convinced that there is a reduction of the EBL. The question was skipped by 23 participants.

For the analysis of EBL, participants were dichotomized into high experience (≥10 procedures per year, n = 49) and low experience (<10 procedures per year, n = 71). Respondents with higher experience were more likely to observe reduced EBL after PE compared to participants with lower experience (OR: 4.55, 95 % CI 1.7–11.9, p = 0.001).

The most commonly reported complications were neurological deterioration due to spinal cord infarction (n = 15), swelling due to tumour necrosis (n = 13), followed by stroke or recent peripheral arterial occlusion (n = 8), hemorrhage (n = 4) and allergic reactions (n = 2). Most participants (72%), however, stated they have not experienced any complication associated with PE taking into account the fact that 20 participants did not answer the question.

## 4 Discussion

In this survey we aimed to investigate the current patterns of care regarding PE for surgery of spinal metastases across Europe. Our results demonstrate that these interventions still appear to be no routine procedures (even at academic hospitals) for more than half of the surgeons that participated in this survey. Interestingly a higher availability of PEs was stated at public, non-university hospitals. Still many patients present as emergencies with neurological compromise and this survey reveals that in those cases PEs are often not requested in order not to delay the emergency surgical treatment.

### 4.1 Rationale for PE

18 % (n = 16) of the respondents declared that there was no evidence regarding the benefit of embolization, 6 % (n = 5) stated that there was still significant intraoperative blood loss despite PE and 14 % (n = 12) that there was an insignificant amount of intraoperative blood loss even without PE. Considering these results, it appears that the evidence for PE is either inconclusive or not well-known in the European spine surgery community.

Although a significant amount of publications exists, which favor PE for hypervascularized metastatic spine tumors (Wahood et al., 2021; Huang et al., 2023; Zhang et al., 2019) and in case of aggressive surgery with en-bloc spondylectomy or vertebrectomy, there are also several studies, which showed no substantial benefit of PE in this context (Kumar et al., 2016; Reitz et al., 2018). The methodology of many of those studies, however, varies substantially and many of them are retrospective case cohort studies. Moreover, it is hard to quantify the extent and effectiveness of PE, hence there is no general angiographic classification, and it is a center-specific variable whether a selective (occlusion of one or several radicular arteries) or super-selective (occlusion of exclusively tumor nourishing vessels) embolization is performed and what kind of embolic agents (coils, polyvinyl alcohol particles, Onyx) are applied. Many studies evaluated patient groups, who received only decompression or separation surgery and those who were treated with a vertebrectomy or en-bloc spondylectomy together, which are different surgical strategies with variable invasiveness and perioperative morbidity that are problematic to lump together in analyses. While PE may be indicated before procedures that involve one or multiple vertebral body resections and replacement, a randomized controlled trial (RCT) found that for posterior decompression with or without instrumentation, PE did not decrease EBL for all tumor types, whereas in hypervascularized lesions EBL was significantly lower (p = 0.041) with 645 mL (SD, 289 mL) in the embolization group and 902 mL (SD, 416 mL) without PE (Clausen et al., 2015). We are not aware of any RCT so far that analyzes whether for more invasive procedures involving anterior column reconstruction PE might be beneficial (Groot et al., 2022). The extent of surgery and the length of the procedure are among the most important variables, which influence the EBL (Rehak et al., 2008). A vertebral body resection, regardless if through anterior or posterior approach, is a comprehensive spine surgery according to the classification of Mirza et al. (2008), which in the presence of a hypervascularized tumor, e.g. renal cell or thyroid carcinoma metastasis, might lead to excessive blood loss with the need of high-volume transfusions and potentially life-threatening conditions (Reitz et al., 2018; Guzman et al., 2005). Therefore, PE is advocated as adjuvant therapy in case of suspected hypervascularized lesions (Guzman et al., 2005; Facchini et al., 2021). PE might improve the surgical outcome through EBL reduction, facilitating complete tumor resection and possibly increasing tumor susceptibility to chemotherapy or radiation therapy (Wahood et al., 2021; Facchini et al., 2021). There is no clear benefit of PE for non-hypervascularized tumors, according to recent data (Clausen et al., 2015; Groot et al., 2022; Yoo et al., 2019). The fact that PE does not reduce EBL for all procedures and tumor types is reflected by the survey results, where 64% of the respondents stated that they observed – or had the impression – that EBL was lower after PE (Fig. 3).

### 4.2 Defining hypervascularity of metastatic tumors

Histopathological findings and CT/MRI imaging have been indicated by most participants as main criteria for decision-making regarding the indication of PE. However, the hypervascularity of metastatic neoplasms is not entirely predictable by histopathology nor MR sequences (Yoo et al., 2019; Prabhu et al., 2003). Further parameters, e.g., tumor volume, extraosseous tumor component and type of surgery are also important features. In addition, preoperative treatment modalities, e.g., chemotherapy and radiotherapy can alter the vascularity of spinal metastases (Huang et al., 2023). A diagnostic angiography displays the tumor vascularity and exposes its relation to the radiculomedullary artery (RMA) at the target level (Huang et al., 2023). Moreover a shared blood supply with the RMA is the most important factor precluding a complete embolization (Prabhu et al., 2003). In cervical spine tumors, PE is usually more difficult than in tumors originating in the thoracic or lumbar spine, due to frequent anastomoses between the carotid, vertebral, and subclavian arteries. The use of additional angiographic assessments to diagnose the vascular structures and the hypervascularity of metastatic spine tumors may be reasonable as diagnostic adjunct (Huang et al., 2023). On the other hand, routine contrast-enhanced MRI might better represent the blood supply from tiny arterial tumor feeders that cannot be selectively interrogated with angiography (Zhang et al., 2019). This makes clear that the sum of all features makes the decision for or against PE more comprehensive.

### 4.3 Optimal time frame

Regarding the optimal timing for PE, most of the participants who declared to perform PE, specified that they plan the intervention <24 h before surgery in a separate anesthesia. Most studies indicate that the time interval between PE and the subsequent spine surgery should be kept as short as possible (<24 h) (Kato et al., 2013; Yuh, 2023; Kumar et al., 2016). Indeed, the grade of embolization and the time interval between PE and surgery were the key factors for EBL reduction in a recent meta-analysis (Yuh, 2023). The duration of the effect of PE is not entirely clear and likely also depends on the embolization agent. The main concerns include the possibilities of either arterial recanalization or the establishment of collateral blood flow. Most surgeons in this survey agreed that the optimal timing for surgery after PE might be “the sooner the better”. This way also the chance of spinal cord compression from post-embolization swelling or hemorrhage might be reduced (Kumar et al., 2016). Most respondents indicated to prefer a separate anesthesia for the PE, which allows for the evaluation of the neurological status after tumor embolization and before subsequent surgery. Complications may occur even with periprocedural neuromonitoring, especially in cases with involvement of the RMA (Yoo et al., 2019). Regardless of the complications, it was evident in our survey that surgeons with greater experience (over 10 procedures per year) were more likely to see the advantages of PE in terms of lower EBL.

### 4.4 Complications of PE

The overall risk for complications in PE has been reported to be on average 3.1 % in a recent systematic review and metanalysis (Griessenauer et al., 2016). Interestingly 28% of all participants in this survey stated that they have experienced procedure-related complications, e.g., neurological deterioration due to spinal cord infarction or due to swelling-related tumor necrosis. Neurologic compromise is a previously described risk of PE for spinal metastasis from either compromise of the spinal cord vascular supply or embolic stroke from reflux of particles (Houten et al., 2020). Postprocedural tumor swelling, however, rarely leads to clinical deficits (Houten et al., 2020). Although these complications have been reported infrequently before, it appears that a considerable number of surgeons have experienced them. This poses the question, whether periprocedural complications are underreported in today's literature. Another possible explanation could be a bias, resulting from experience. Our survey comprised relatively few participants from high-volume centers. The number and variety of complications might be lower in those institutions, however, since there is higher level of experience. We cannot fully exclude that survey participants reported complications that they had heard of, but not experienced in their own patients; this would also explain a relatively high rate of reported complications.

### 4.5 Strengths and limitations

This survey provides an overview on the current state of PE for spine metastasis across European spine surgeons. The data can serve as a reference for further studies and questions in this context. The survey gives a first impression of the subjective evaluation of the utilization of PE, which appears to match several results and considerations from prior cohort studies, systematic reviews and meta-analysis.

The limitations of this work are the way of data collection, which requires voluntary, non-incentivized participation and subjective evaluation. These requirements lead to a possible selection bias. Furthermore, a low survey response rate in general, and relatively low participation from participants working at high-volume centers must be disclosed. The survey participants were mostly neurosurgeons from European countries, which further limits the generalization of these findings to other regions and settings.

## 5 Conclusions

PE for spinal metastases appears to be a non-routine practice among European spine surgeons today. In cases with suspected hypervascularized tumor types, without acute neurological deterioration requiring urgent surgery, most surgeons prefer to perform PE in a separate anesthesia <24h before subsequent surgical treatment. The majority of participants in this study stated a reduction of EBL but also a non-negligible rate of observed procedure-related complications.

## Declaration of competing interest

The authors declare that they have no known competing financial interests or personal relationships that could have appeared to influence the work reported in this paper.
