# Supplementary material for: Current state of preoperative embolization for spinal metastasis – A survey by the EANS spine section
Source: Brain Spine. 2023 Nov 10;3:102712. doi: 10.1016/j.bas.2023.102712 (PMC10668085; doi:10.1016/j.bas.2023.102712)
Supplement: Multimedia component 1 [file mmc1.docx]

**Supplemental Material**

**Nr. 1.** **Survey questions**

Demographic information:

1) Age: _________ years

2) Gender: (single answer)

a. Male

b. Female

3) Education level: (single answer)

a. Specialist in neurosurgery

b. Specialist in orthopedics

c. Specialist in trauma surgery/traumatology

d. Resident in advanced training

e. Other: _______________________

4) Workplace (single answer)

a. University Hospital

b. Public, non-university hospital

c. Private hospital

d. Medical practice

e. Other: __________________________

5) Average number of spinal stabilization procedures performed by you personally for vertebral metastases per year (single answer)

a. < 5

b. 5 - 10

c. 10 - 20

d. 20 - 30

e. > 30

Survey:

6) Do you routinely perform preoperative embolizations in selected patients with spinal metastases? (Single answer)

a. Yes, but only when vertebral body resection/replacement is necessary

b. Yes, in general before any elective surgical treatment (including tumor decompression alone, "separation surgery" without vertebral body replacement, etc.).

c. No

7) If yes, for metastases of which tumor type do you perform preoperative embolization? (Multiple answers possible)

a. For hypervascularized tumors, e.g., renal cell carcinoma or thyroid carcinoma

b. For recurrent surgery or pre-radiated situs (surgical approach less clear/generous)

c. In general for all dignities

d. We do not perform preoperative embolization at all

8) If no, why do you not routinely perform preoperative embolizations in patients with spinal metastases? (Multiple answers possible)

a. There is no clear evidence regarding benefit

b. There is a significant amount of bleeding despite embolization

c. It does not bleed much even without embolization

d. There is no possibility for preoperative embolization in our hospital

e. We usually operate on emergency cases (with acute neurological deficits), where there is no time for embolization

f. Other reasons: ____________________

9) Which criteria do you use to make the indication for preoperative embolization? (Multiple answers possible)

a. Preoperative MRI and/or CT

b. Preoperative MR angiography or CT angiography

c. Diagnostic subtraction angiography

d. (Suspected) tumor histopathology

e. Preoperative (tumor) anemia / anticoagulation status

f. Other reasons: _________________________

g. We do not perform embolizations

10) At what time interval are preoperative embolizations for subsequent surgery preferentially performed in your practice? (Single answer)

a. Immediately before surgery (same anesthesia)

b. Within 24 h before surgery

c. Within 48 h before surgery

d. Within 72 h or more before surgery

e. We do not perform embolizations

11) Do you have the impression, or have you evaluated, that intraoperative blood loss is lower due to preoperative embolizations in your practice? (Single answer)

a. Yes

b. No

12) Have you experienced complications from preoperative embolizations? (Multiple answers possible)

a. Yes, neurologic deterioration due to swelling-related tumor necrosis

b. Yes, neurologic deterioration due to spinal cord infarction (occlusion of segmental radicular artery, Adamkiewicz artery, etc.)

c. Yes, stroke or acute peripheral arterial occlusion due to catheter-associated embolization

d. Yes, allergic reaction to embolization material

e. Yes, catheter-associated hemorrhage

f. Yes, other: __________________

g. No, none

Participation:

13) My participation in this survey is:

a. Anonymous

b. By name, and I wish to be listed in the Appendix of the article / Pubmed: _________________________ (please ensure correct form of named information: "last name, first name").

**Nr. 2 List of contributors**

| **Name** |
| --- |
|  |
| Uribe-Pacheco, Rodrigo |
| Gadjradj, Pravesh |
| Snopko, Pavol |
| Busch, Stefan |
| Chihi, Mehdi |
| Obid, Peter |
| Platz, Uwe |
| Schwake, Michael |
| Bludau, Frederic |
| Demetriades, Andreas K. |
| Harel, Ethan |
| Maldaner, Nicolai |
| Ilić, Jovan |
| Adrian, Thomas May |
| Klingler, Jan-Helge |
| Thijs, Dieter |
| Troude, Lucas |
| Lener, Sara |
| Santhosh, Isaac Poonoose |
| Bonk, Maximilian-Niklas |
| Rasschaert, Ricky |
| Giamundo, Marialaura |
| Capo, Gabriele |
| Santos, Edgar |
| Schär, Ralph T. |
| Colasanti, Roberto |
| Sliauzys, Albertas |
| Boukebir, Mohamed Abdelatif |
| Montemurro, Nicola |
| Hamouda, Waeel |
| Miljković, Aleksandar |
| Syrmos, Nikolaos |
| Lubrano, Vincent |
| Vajkoczy, Peter |
| Georgiopouls, Miltiadis |
| Trnovec, Svorad |
| Gankpe, Gbètoho Fortuné |
| Chrenko, Robert |
| João, Pedro Oliveira |
| Trnovec, Benedikt |
| Pravesh, Gadjradj |
| Nunes de Sequeira, Sara |
| Lofrese Giorgio |
| Ihor, Kotskovych |
| Butenschoen, Vicki M. |
| Farag, Ahmed |
| Cortier, Jeroen |
| Ievgenii Iarmoliuk |
| Stogowski, Piotr |
| Meyer, Bernhard |
| Brinyuk, Evgeny |
| Navarro, Ramon |
| Lombard, Arnaud |
| Plata-Bello, Julio |
| Visocchi, Massimiliano |
| Sistiaga, Iñigo L. |
| Amoo, Michael |
| Jolayemi, Edward |
| Sagerer, Andre |
| Cornelius, Jan Frederick |
| Charitos, Dimitrios |
| Rahman, Moshiur |
| Vankipuram, Siddharth |
| Auslands, Kaspars |
| Marchi, Francesco |
| Georgios, Alexiou |
| Golubovic, Jagos |
| Depreitere, Bart |
| Ziga, Michael |
| Lawson McLea, Aaron |
| Carretta, Alessandro |
| Jankovic, Dragan |
| Aftahy, Amir Kaywan |
